# Faculty adoption of AI-assisted teaching tools in Chinese higher education: an integrated UTAUT2–TAM framework at Shanghai University

**DOI:** 10.3389/frai.2026.1847439

**Published:** 2026-05-29

**Authors:** Jie Gao, Wenshuo Yang

**Affiliations:** 1 SILC Business School, Shanghai University, Shanghai, China; 2 Science and Technology Industry Research Center, Law School, Shanghai University, Shanghai, China

**Keywords:** AI-assisted teaching, artificial intelligence, Chinese universities, faculty attitudes, higher education, PLS-SEM, TAM, technology adoption

## Abstract

**Introduction:**

This study tests an integrated UTAUT2–TAM framework for faculty adoption of AI-assisted teaching tools at Shanghai University.

**Methods:**

A mixed-methods sequential explanatory design combined a quantitative survey of 326 faculty members with 22 semi-structured interviews. PLS-SEM, MICOM, PLS-MGA, and codebook thematic analysis were used.

**Results:**

Performance expectancy, perceived usefulness, institutional support, social influence, and AI self-efficacy were the strongest positive correlates of faculty attitude, while perceived risk showed a significant negative association. The model explained 62.1% of variance in attitude, 52.7% in adoption intention, and 48.3% in self-reported adoption behavior.

**Discussion:**

The findings demonstrate discipline-specific adoption pathways and identify compliance-driven adoption among senior faculty as a boundary condition for the attitude-mediation mechanism.

## 1 Introduction

Generative AI platforms, intelligent tutoring systems, AI-powered grading tools, and adaptive learning environments have entered routine use in higher education over the past 5 years (Zawacki-Richter et al., 2019; Holmes et al., 2022; Kasneci et al., 2023). In China, two national policies have accelerated institutional uptake: the New Generation Artificial Intelligence Development Plan (State Council of the PRC, 2017) and the Education Informatization 2.0 Action Plan (Ministry of Education of the PRC, 2018). Shanghai University, located in a municipality designated as a pilot zone for AI-enabled education (Shanghai Municipal Education Commission, 2021), has procured AI-based learning platforms, launched faculty training initiatives, and encouraged integration of AI tools in undergraduate teaching (Shanghai University, 2023). Despite these investments, actual adoption among faculty remains uneven—a pattern consistent with findings in both Chinese and international contexts (Crompton and Burke, 2023; Wang et al., 2021).

Decades of research on technology acceptance have shown that the availability of technology does not guarantee its use; adoption is mediated by individual beliefs, attitudes, social norms, organizational support, and characteristics of the technology itself (Davis, 1989; Venkatesh et al., 2003; Rogers, 2003). AI tools differ from earlier educational technologies in the scope of their functional overlap with core faculty work: large language models can draft content, grade open-ended assignments, and interact with students conversationally—activities previously regarded as core pedagogical labor (Chan and Hu, 2023; Baidoo-Anu and Ansah, 2023). Faculty therefore face adoption decisions that are not only instrumental but also evaluative, with concerns about academic integrity, pedagogical authority, and professional identity reported alongside efficiency gains (Moorhouse et al., 2023; Chan and Hu, 2023).

Most empirical studies on AI in higher education focus on students—their attitudes, learning outcomes, and ethical concerns (Chan and Hu, 2023; Crompton and Burke, 2023). Faculty, as the primary agents of pedagogical integration, have received comparatively less attention, and the gap is especially pronounced for Chinese research universities specifically (Scherer et al., 2019; An et al., 2023). Studies that do address faculty adoption rely predominantly on single frameworks—TAM (Davis, 1989) or UTAUT (Venkatesh et al., 2003)—applied without AI-specific constructs such as perceived risk and AI self-efficacy, and without verifying measurement invariance before making disciplinary or rank-based subgroup comparisons (Granić and Marangunić, 2019; An et al., 2023). The methodological gap is therefore threefold: (a) no published study has tested a UTAUT2–TAM integration augmented with AI-specific constructs in a Chinese Tier-1 research university faculty sample; (b) no prior study in this domain has conducted MICOM-validated multi-group analysis across disciplinary clusters to test whether the structural paths are comparably measured before claiming subgroup differences; and (c) no mixed-methods study has used a formal joint-display framework to identify integration patterns—including divergent findings—between quantitative adoption models and qualitative accounts.

This study addresses all three gaps. Three research questions guide the analysis:

*RQ1*: Which UTAUT2, TAM, and AI-specific constructs are associated with faculty attitude toward AI-assisted teaching tools at Shanghai University, and with what relative strength?

*RQ2*: To what extent does faculty attitude mediate the paths from perceived usefulness and perceived ease of use to adoption intention, and does PEOU also operate indirectly through PU, consistent with the TAM chain?

*RQ3*: How do teaching discipline, academic rank, and prior technology experience moderate the structural paths, and do the qualitative interviews converge with, expand on, clarify, or diverge from these subgroup differences?

The study contributes on three fronts: a MICOM-validated multi-group demonstration that disciplinary culture conditions AI adoption pathways; a qualitative boundary condition for the attitude-mediation mechanism; and actionable institutional-policy levers for AI-teaching integration in Chinese higher education. The remainder of the paper is structured as follows: Section 2 reviews the literature; Section 3 develops hypotheses; Section 4 describes methods; Section 5 reports results; Section 6 discusses implications; Section 7 concludes.

## 2 Literature review

### 2.1 Artificial intelligence in higher education: global trends and the Chinese context

AI applications in higher education have evolved from rule-based expert systems in the 1980s and 1990s to the current generation of foundation models capable of natural-language understanding, code generation, and multimodal reasoning (Holmes et al., 2022). Zawacki-Richter et al. (2019) organise the field into four application areas: adaptive and personalized learning, profiling and prediction, assessment and evaluation, and intelligent tutoring systems. The release of large language models such as GPT-4 and their Chinese counterparts (Baidu’s ERNIE Bot, Alibaba’s Tongyi Qianwen) has spurred a second wave of experimentation, with faculty exploring applications in lecture preparation, automated grading, and real-time student feedback (Kasneci et al., 2023; Baidoo-Anu and Ansah, 2023).

In China, AI in education has been shaped by a combination of state direction and private-sector innovation. The New Generation AI Development Plan (State Council of the PRC, 2017) positioned AI-enabled education as a strategic priority, and the Education Informatization 2.0 Action Plan (Ministry of Education of the PRC, 2018) provided operational guidelines. At the municipal level, Shanghai has invested heavily in smart-campus infrastructure (Shanghai Municipal Education Commission, 2021). Within this environment, Shanghai University has implemented AI-assisted platforms including intelligent course-recommendation systems and virtual teaching assistants (Shanghai University, 2023). A recurring finding in both international and Chinese literature, however, is that institutional investment in AI infrastructure does not automatically translate into widespread faculty adoption (Crompton and Burke, 2023; Wang et al., 2021). Faculty responses range from enthusiastic early adoption through cautious experimentation to outright resistance.

### 2.2 Faculty adoption of educational technology

A meta-analysis by Scherer et al. (2019) of 114 studies—fewer than 20% of which focused exclusively on faculty—identifies perceived usefulness, perceived ease of use, subjective norms, and self-efficacy as the most consistent predictors of faculty technology acceptance. Teo (2011) shows these factors operate similarly across cultural contexts, including East Asian settings, although social influence carries more weight in collectivist cultures. Studies in Chinese and Hong Kong contexts have confirmed these patterns while highlighting the role of institutional incentive structures, administrative mandates, and technical-support availability (Wang and Cheng, 2021; Chatterjee and Bhattacharjee, 2020).

For AI tools specifically, faculty attitudes appear more ambivalent than for earlier technologies. Moorhouse et al. (2023) find that faculty in Hong Kong value efficiency gains offered by AI-assisted grading and content generation but express concerns about the erosion of pedagogical autonomy. Chan and Hu (2023) document similar findings in mainland China, with academic integrity and reliability of AI-generated content as significant barriers. Wang et al. (2021) demonstrate that attitude toward AI mediates the effects of self-efficacy, perceived ease of use, and perceived usefulness on faculty adoption intention in Chinese and Taiwanese higher education. An et al. (2023) extend this to a Chinese middle-school EFL context using UTAUT combined with domain-specific knowledge. Together, these studies indicate that traditional technology-acceptance models require augmentation with AI-specific constructs.

### 2.3 Technology acceptance model (TAM) and its extensions

The Technology Acceptance Model (TAM; Davis, 1989) posits that perceived usefulness (PU) and perceived ease of use (PEOU) determine attitude toward a technology, which in turn influences behavioral intention and actual use. TAM has been among the most widely applied frameworks in educational-technology research (Granić and Marangunić, 2019). TAM has also been criticised for neglecting social, organizational, and contextual factors (Bagozzi, 2007). Extensions include TAM2 (Venkatesh and Davis, 2000), which incorporates social-influence processes, and TAM3 (Venkatesh and Bala, 2008), which integrates determinants of PEOU. Despite these extensions, critics have argued that TAM remains limited by its emphasis on individual-level beliefs at the expense of structural and institutional factors (Straub, 2009).

### 2.4 Unified theory of acceptance and use of technology (UTAUT/UTAUT2)

UTAUT (Venkatesh et al., 2003) synthesizes constructs from eight technology-acceptance theories including TAM, the Theory of Planned Behavior (Ajzen, 1991), Innovation Diffusion Theory (Rogers, 2003), and Social Cognitive Theory (Bandura, 1986). It posits four core determinants: performance expectancy (PE), effort expectancy (EE), social influence (SI), and facilitating conditions (FC), with gender, age, experience, and voluntariness as moderators. UTAUT2 (Venkatesh et al., 2012) extended the model for consumer contexts by adding hedonic motivation, price value, and habit. The broader UTAUT framework’s explicit treatment of social and organizational factors makes it well suited to institutional settings (Dwivedi et al., 2019). Empirical applications in higher education generally support its predictive validity, with PE and FC as the strongest predictors of faculty technology use (Khechine et al., 2020).

### 2.5 Integrating TAM and UTAUT2: rationale for a hybrid model

TAM and UTAUT have historically been treated as competing alternatives, but a growing literature argues for integration on the grounds that the two models capture complementary aspects of the adoption process (Dwivedi et al., 2019; Granić and Marangunić, 2019). TAM’s strength is a parsimonious account of individual cognitive beliefs (PU, PEOU) and their relationship to attitude—a construct that UTAUT deliberately excluded for parsimony but that retains substantial predictive power (Dwivedi et al., 2019). UTAUT’s strength is a broader treatment of social influence, facilitating conditions, and moderating variables that TAM underspecifies. An integrated UTAUT2–TAM framework therefore retains TAM’s attitude-mediation pathway while incorporating UTAUT2’s structural and social determinants.

This integration is especially warranted for AI adoption, where both individual cognitive appraisals (Is this tool useful? Is it easy to use?) and institutional factors (Does my university support this? Do my colleagues use it?) shape the decision (Scherer et al., 2019). As specified in the methodological gap in 1, no prior study has applied such an integrated model with MICOM-validated multi-group analysis in a Chinese Tier-1 research university faculty context.

### 2.6 Conceptual distinction between PE/PU and between FC/IS

Because the proposed model retains both performance expectancy (PE) from UTAUT2 and perceived usefulness (PU) from TAM, and both facilitating conditions (FC) from UTAUT2 and institutional support (IS) as an AI-specific construct, each pair requires explicit differentiation to justify their simultaneous inclusion.

PE vs. PU. PE captures the role-level judgement that AI tools help faculty perform their teaching role more effectively, with items referencing lecture preparation, grading, and feedback as outcomes (Venkatesh et al., 2003). PU captures the task-level judgement that AI tools are instrumentally useful for specific teaching tasks such as content generation and question construction (Davis, 1989). The relationship is therefore hierarchical: PU is proximal to task-level usage; PE is distal to role-level performance. The TAM tradition emphasises the attitude-mediation pathway (PU → ATT → intention), whereas UTAUT models PE as a direct antecedent of intention. Retaining both tests whether task-level and role-level utility beliefs contribute independent variance to attitude. Empirical discriminant validity is confirmed: HTMT (PE, PU) = 0.782, below the 0.85 threshold.

FC vs. IS. FC captures faculty perceptions of the objective organizational and technical infrastructure enabling AI use—IT support, training availability, compatible systems, and time allocation (Venkatesh et al., 2003). IS is broader and more normative: it encompasses the university’s provision of training, incentives, policy guidance, and recognition of AI-enhanced teaching in promotion criteria (Wang and Cheng, 2021). FC thus captures the hardware and logistics of AI use, whereas IS captures symbolic and normative institutional signals. In the Chinese higher-education system, where institutional signals strongly shape faculty priorities, this normative layer is expected to have significant predictive power beyond FC. Consistent with this operationalisation, FC is modelled as a direct antecedent of adoption behavior (infrastructural enablers translate intention into action) whereas IS is modelled as an antecedent of attitude (the institutional signal shapes evaluative orientation). HTMT (FC, IS) = 0.714, confirming empirical distinctness.

### 2.7 Attitudes, AI self-efficacy, and perceived risk

Three additional constructs are of particular relevance to faculty AI-tool adoption.

Attitude toward AI has been identified as a central mediator in technology adoption, particularly when the technology is perceived as transformative or potentially disruptive (Dwivedi et al., 2019). Unlike routine educational technologies, AI tools can generate content, make evaluative judgments, and interact with students in ways that directly overlap with traditional faculty roles, making attitudinal orientation a critical determinant of adoption willingness (Moorhouse et al., 2023).

AI self-efficacy, defined as an individual’s belief in the ability to use AI-based tools effectively for teaching purposes, extends the general computer-self-efficacy construct (Compeau and Higgins, 1995) to the domain of artificial intelligence. Research on digital self-efficacy in educational contexts has consistently shown that confidence in one’s ability to use technology predicts adoption independently of the technology’s objective ease of use (Scherer et al., 2019; Wang et al., 2021).

Perceived risk encompasses faculty concerns about potential negative consequences of AI adoption, including threats to academic integrity, loss of pedagogical control, data-privacy issues, algorithmic bias, and reliability of AI-generated content (Chan and Hu, 2023; Moorhouse et al., 2023). While perceived risk has been studied in consumer technology adoption (Featherman and Pavlou, 2003), Chiu et al. (2023) provide a more contextually relevant taxonomy of AI-specific risk dimensions in higher education, distinguishing among performance risk, privacy risk, integrity risk, and autonomy risk. This taxonomy is reflected in the five-item PR scale used in the present study (4.3.1). Its application to AI-assisted teaching—and specifically to faculty adoption decisions—remains limited, and the inclusion of PR in the proposed framework responds to calls in the literature for technology-acceptance models to account for the uncertainties associated with emerging AI technologies (Featherman and Pavlou, 2003; Chiu et al., 2023).

## 3 Theoretical framework and hypotheses

### 3.1 Proposed integrated model

Drawing on the literature above, the integrated UTAUT2–TAM model incorporates nine independent constructs across three layers: (a) core UTAUT2 constructs (PE, EE, SI, FC); (b) core TAM constructs (PU, PEOU); and (c) AI-specific constructs [AI self-efficacy (AISE), perceived risk (PR), institutional support (IS)]. Faculty attitude toward AI (ATT) mediates between these antecedents and adoption intention (AI-Intent), which in turn predicts self-reported adoption behavior. Facilitating conditions additionally predict adoption behavior directly. Three moderating variables—teaching discipline, academic rank, and prior technology experience—are hypothesized to condition the strength of key structural paths, and are examined via PLS-MGA following MICOM invariance testing. Conceptual distinctions between PE and PU and between FC and IS are specified in 2.6.

### 3.2 Hypothesis development

Hypotheses are worded as predicted associations rather than causal claims, consistent with the cross-sectional design.

*H1*: Performance expectancy is positively associated with faculty attitude toward AI-assisted teaching tools. Faculty who perceive AI tools as capable of improving teaching efficiency, student engagement, or assessment quality are expected to develop more favorable attitudes (Venkatesh et al., 2003; Khechine et al., 2020).

*H2*: Effort expectancy is positively associated with faculty attitude. Faculty who perceive AI tools as requiring minimal additional effort are more likely to form positive attitudes, particularly in early adoption stages (Venkatesh et al., 2003).

*H3*: Social influence is positively associated with faculty attitude. In the collectivist cultural context of Chinese higher education, where collegial norms and institutional expectations exert strong influence on individual behavior, social influence is expected to be a particularly salient predictor (Teo, 2011; An et al., 2023).

*H4*: Facilitating conditions are positively associated with self-reported adoption behavior. FC reflects the objective enablers and constraints determining whether intentions can translate into action (Venkatesh et al., 2003, 2012).

*H5*: Perceived usefulness is positively associated with faculty attitude. PU captures task-level beliefs about the instrumental value of AI tools; the attitude-mediation pathway explains how utility beliefs translate into adoption through attitudinal change (Davis, 1989; Granić and Marangunić, 2019).

*H6*: Perceived ease of use is positively associated with faculty attitude, and additionally exerts an indirect effect on attitude via perceived usefulness (the TAM chain PEOU → PU → ATT), tested with VAF (Davis et al., 1989; Wang et al., 2021).

*H6a*: Perceived ease of use is positively associated with perceived usefulness. Technologies perceived as easier to use are also judged as more useful, because reduced effort frees cognitive resources for recognising productive benefits (Davis, 1989; Davis et al., 1989).

*H7*: AI self-efficacy is positively associated with faculty attitude. Faculty with higher AISE approach AI tools with greater confidence and less anxiety, leading to more favorable attitudes (Compeau and Higgins, 1995; Scherer et al., 2019).

*H8*: Perceived risk is negatively associated with faculty attitude. Higher PR—regarding academic integrity, pedagogical control, data privacy, algorithmic bias, or reliability—is associated with negative attitudes toward AI tools (Featherman and Pavlou, 2003; Chiu et al., 2023; Chan and Hu, 2023).

*H9*: Institutional support is positively associated with faculty attitude. Beyond UTAUT’s FC, IS captures symbolic and normative institutional signals that strongly shape faculty priorities in the Chinese higher-education system (Wang and Cheng, 2021).

*H10*: Faculty attitude toward AI mediates the relationships between the antecedent constructs (H1–H3, H5–H9) and adoption intention. Attitude serves as the integrative psychological mechanism through which diverse cognitive appraisals and social perceptions are synthesized into an evaluative orientation (Ajzen, 1991; Dwivedi et al., 2019).

*H11*: Adoption intention is positively associated with self-reported adoption behavior (Venkatesh et al., 2003, 2012).

## 4 Materials and methods

### 4.1 Research design

The study employed a mixed-methods sequential explanatory design (Creswell and Plano Clark, 2018; Tashakkori and Teddlie, 2010), in which a quantitative survey phase was followed by a qualitative interview phase. The quantitative phase tested the hypothesized relationships across a representative faculty sample. The qualitative phase was informed by the quantitative findings and designed to elicit mechanisms and institutional dynamics underlying the observed patterns. Integration of the two strands followed the joint-display framework of Fetters et al. (2013), with convergence, expansion, clarification, and divergence as the four integration patterns reported in 5.9.

### 4.2 Participants and sampling

The target population consisted of full-time faculty members at Shanghai University with undergraduate teaching responsibilities. Shanghai University employs approximately 3,000 full-time faculty across more than 70 departments organized into four disciplinary clusters: STEM, Social Sciences, Humanities, and Arts. A stratified random sampling strategy ensured proportional representation across clusters, with oversampling of smaller clusters (Humanities, Arts) to support multi-group analysis.

A total of 380 questionnaires were distributed; 341 were returned (response rate: 89.7%). After removing 15 incomplete responses (more than 10% missing data handled by list-wise exclusion), 326 valid responses were retained. This sample exceeds the minimum requirement for PLS-SEM of 10 times the maximum number of structural paths directed at any single construct (Hair et al., 2019). With nine paths directed at the mediator (ATT), the minimum requirement was 90; the achieved sample of 326 provided substantial headroom for subgroup analyses.

For the qualitative phase, 22 faculty members were selected through purposive maximum-variation sampling to ensure representation across disciplines (STEM: 7; Social Sciences: 6; Humanities: 5; Arts: 4), academic ranks (lecturers: 7; associate professors: 8; professors: 7), and levels of AI-tool adoption (non-adopters: 6; occasional users: 9; regular users: 7). Subgroup sizes for the three MGA contrasts were: STEM *n* = 117 vs. Humanities *n* = 70; lecturers *n* = 103 vs. full professors *n* = 95; low-experience *n* = 71 vs. high-experience *n* = 107.

### 4.3 Instruments

#### 4.3.1 Quantitative survey instrument

The structured questionnaire comprised three sections: (a) demographic and professional background; (b) AI-tool awareness and usage patterns; and (c) measurement scales for the nine independent constructs, the mediator, and the dependent variables. All attitudinal and perceptual items used a 7-point Likert scale (1 = strongly disagree, 7 = strongly agree). Scales were adapted from established instruments with AI-specific item wording. The five-item PR scale captures five facets of AI risk—academic integrity, autonomy loss, data privacy, algorithmic bias, and reliability—consistent with Chiu et al.'s (2023) taxonomy. Table 1 summarises the constructs, sources, and sample items. The full questionnaire item list is provided in the supplementary materials to support replication.

**Table 1:** Construct definitions, scale sources, and sample items.

Construct	Source	Items	Sample item
Performance expectancy (PE)	Venkatesh et al. (2003)	4	Using AI teaching tools increases my productivity in course preparation.
Effort expectancy (EE)	Venkatesh et al. (2003)	4	Learning to use AI teaching tools is easy for me.
Social influence (SI)	Venkatesh et al. (2003)	4	Colleagues whose opinions I value think I should use AI teaching tools.
Facilitating conditions (FC)	Venkatesh et al. (2003)	4	I have the resources necessary to use AI teaching tools.
Perceived usefulness (PU)	Davis (1989)	4	Using AI teaching tools would improve my teaching effectiveness.
Perceived ease of use (PEOU)	Davis (1989)	4	I find AI teaching tools easy to use for course design.
AI self-efficacy (AISE)	Compeau and Higgins (1995), adapted	5	I am confident I can effectively integrate AI tools into my teaching.
Perceived risk (PR)	Featherman and Pavlou (2003), Chiu et al. (2023), adapted	5	I worry about academic-integrity issues when students use AI tools.
Institutional support (IS)	Wang and Cheng (2021), adapted	4	My university provides adequate training for AI-integrated teaching.
Attitude toward AI (ATT)	Ajzen (1991), adapted	4	Using AI teaching tools is a good idea.
Adoption intention (AI-intent)	Venkatesh et al. (2003)	3	I intend to use AI teaching tools in the next academic semester.

The questionnaire was developed initially in English, then translated into Mandarin Chinese using a back-translation procedure (Brislin, 1970). A bilingual research panel reviewed the translated instrument for semantic and cultural appropriateness. Prior to full deployment, the instrument was pilot-tested with 45 faculty members (all constructs: Cronbach’s *α* = 0.82–0.91). Minor wording adjustments were made based on respondent feedback.

#### 4.3.2 Qualitative interview protocol

The semi-structured interview protocol comprised six guiding questions: (1) experience with AI-assisted teaching tools and contexts of use; (2) factors influencing the adoption decision; (3) departmental and university support structures; (4) concerns about AI use in teaching; (5) perceived impact on student learning outcomes and academic integrity; and (6) conditions that would facilitate future adoption. Interviews lasted 35–55 min (mean: 43 min).

### 4.4 Data collection procedure

Ethical approval was obtained from the Shanghai University Institutional Review Board prior to data collection [protocol reference SHU-IRB-2025 (number to be confirmed on submission)]. All participants provided written informed consent. The quantitative survey was administered online via Wenjuanxing between March and April 2025; faculty were recruited through departmental email lists with follow-up reminders at two-week intervals. Participation was voluntary, with a 50 RMB gift card offered as an incentive. Interviews were conducted in Mandarin Chinese between May and June 2025, audio-recorded with participant consent, and transcribed verbatim.

### 4.5 Data analysis

#### 4.5.1 Quantitative analysis

PLS-SEM was implemented in SmartPLS 4 (Ringle et al., 2022). PLS-SEM was chosen over covariance-based SEM because (a) it is well suited to exploratory theory-building research with complex models (Hair et al., 2019); (b) it performs reliably at the achieved sample size without requiring multivariate-normal indicators; and (c) it handles multiple mediating and moderating relationships (Venkatesh et al., 2012). The measurement model was assessed for internal consistency reliability (Cronbach’s *α*, composite reliability), convergent validity (AVE), and discriminant validity (HTMT, Fornell–Larcker). The structural model was assessed by path coefficients (*β*), *R*^2^, effect sizes (*f*^2^), and predictive relevance (*Q*^2^) via blindfolding. Mediation effects, including the PEOU → PU → ATT chain with VAF, were tested using bootstrapping (5,000 resamples, Hair et al., 2019). Common method bias was assessed via Harman’s single-factor test and inner VIF (Kock, 2015). Measurement invariance was established using MICOM (Henseler et al., 2016) before PLS-MGA; the procedure and results are reported in 4.6 and 5.6.

#### 4.5.2 Qualitative analysis (codebook thematic analysis)

Interview transcripts were analyzed using codebook thematic analysis, drawing on established procedures for thematic coding and code development (Boyatzis, 1998; Guest et al., 2012; King, 2012), a structured variant of thematic analysis that is designed to be compatible with formal inter-coder reliability indices. This choice is deliberate: reflexive thematic analysis (Braun and Clarke, 2019, 2021) explicitly rejects inter-coder reliability calculations as inconsistent with its constructivist epistemological stance; because the present study reports Cohen’s *κ*, codebook TA is the methodologically consistent designation.

An initial a-priori codebook was developed directly from the nine UTAUT2–TAM constructs (PE, EE, SI, FC, PU, PEOU, AISE, PR, IS) and their relationships to ATT, intention, and behavior. The codebook contained 22 first-level codes at the point of initial deployment. After independent coding of the first five transcripts (22.7% of the corpus) by both researchers, iterative review identified three additional inductive codes not anticipated by the a-priori structure: (i) symbolic vs. substantive support distinction (within IS), (ii) professional-identity threat (a facet of PR not captured by the five PR items as worded), and (iii) compliance-driven adoption without attitudinal alignment. This third inductive code is the basis for Theme 6 (5.8). It emerged exclusively from the data—it was not part of the a-priori UTAUT2–TAM codebook—which is the basis on which it is claimed as a divergent finding in the joint display (5.9). Inter-coder reliability on the five pilot transcripts reached Cohen’s *κ* = 0.84 (Landis and Koch, 1977, strong agreement). Coding continued for all 22 transcripts; no new primary themes emerged after the 19th interview, indicating sufficient thematic stability at the achieved sample size. Analysis was performed in NVivo 14.

### 4.6 Measurement invariance (MICOM) prior to multi-group analysis

Measurement invariance of composites (MICOM) was established before interpreting PLS-MGA findings, following the three-step procedure of Henseler et al. (2016) for three focal contrasts: (A) STEM (*n* = 117) vs. Humanities (*n* = 70); (B) lecturers (*n* = 103) vs. full professors (*n* = 95); (C) low prior technology experience (*n* = 71) vs. high prior technology experience (*n* = 107). Step 1 (configural invariance) holds by construction: identical indicator sets, data treatment, coding, and algorithm settings were applied across all subgroups. Step 2 (compositional invariance) was assessed using permutation tests (5,000 permutations) on the composite correlations between subgroups. Step 3 (equality of composite means and variances) was assessed using permutation-based 95% confidence intervals. Full MICOM results are reported in 5.6 (Table 2). All three contrasts achieved full measurement invariance; PLS-MGA results are therefore interpretable as genuine differences in structural path strength rather than measurement artefacts.

**Table 2:** Demographic profile of respondents (*N* = 326).

Variable	Category	*n*	%
Gender	Male	176	54.0
	Female	150	46.0
Academic rank	Lecturer	103	31.6
	Associate Professor	128	39.3
	Professor	95	29.1
Discipline	STEM	117	35.9
	Social Sciences	89	27.3
	Humanities	70	21.5
	Arts	50	15.3
Prior tech experience	Low	71	21.8
	Medium	148	45.4
	High	107	32.8
AI adoption (past year)	Non-adopter	169	51.8
	Adopter (any use)	157	48.2

### 4.7 Ethics and researcher reflexivity

This study involved human participants and was classified as human-subjects research. Ethical approval was granted by the Shanghai University Institutional Review Board. The study adhered to the ethical principles of the Declaration of Helsinki and complied with Shanghai University’s research-ethics regulations. Safeguards included voluntary participation with the right to withdraw, written informed consent, anonymisation of survey responses, pseudonymisation of interview transcripts, and secure storage on encrypted university servers. The first author is a faculty member at the SILC Business School of the institution studied. This insider position facilitated recruitment but introduced potential bias, managed through four mechanisms: (i) a non-insider co-investigator (second author, Law School) independently coded qualitative transcripts; (ii) interview transcripts were not shared with participants’ immediate line managers; (iii) interview quotes were pseudonymised before analysis; and (iv) themes were reviewed across disciplinary subgroups to avoid over-representing the first author’s disciplinary norms.

## 5 Results

### 5.1 Respondent demographics

Table 2 presents the demographic profile of the 326 valid respondents. The sample was balanced on gender (male: 54.0%; female: 46.0%) and academic rank (lecturers: 31.6%; associate professors: 39.3%; professors: 29.1%). Respondents were distributed across STEM (35.9%), Social Sciences (27.3%), Humanities (21.5%), and Arts (15.3%). Mean age was 42.6 years (SD = 9.1); mean teaching experience was 14.2 years (SD = 8.3). Prior technology experience was self-rated as low by 21.8%, medium by 45.4%, and high by 32.8% of respondents—classified on a three-point scale included in the survey instrument. These demographics are broadly consistent with the full-time faculty profile at Shanghai University (Shanghai University, 2023).

Regarding AI-tool awareness, 92.3% (*n* = 301) reported awareness of at least one relevant tool. Actual adoption rates were substantially lower: 48.2% (*n* = 157) reported using any AI-assisted teaching tool in the past academic year. Among adopters, the most common applications were content generation and lecture preparation (41.4%), automated grading and feedback (29.9%), and AI-chatbot student query handling (18.5%).

### 5.2 Common method bias assessment

Harman’s single-factor test on all measurement items yielded a first-unrotated-factor variance of 28.6%, below the 50% threshold (Podsakoff et al., 2003). Inner VIF values ranged from 1.18 to 2.67, below the conservative threshold of 3.3 (Kock, 2015). These indicators suggest that common method bias is unlikely to be a dominant concern, though some residual method variance cannot be excluded given the single-source self-report design (see Section 6.4).

### 5.3 Measurement model assessment

As shown in Table 3, all constructs demonstrated strong internal consistency (Cronbach’s *α* = 0.831–0.912; CR = 0.878–0.935), exceeding the 0.70 threshold (Nunnally, 1978; Hair et al., 2019). AVE values ranged from 0.614 to 0.762, all exceeding 0.50. All outer loadings exceeded 0.70 except PR5 (0.694), retained given near-threshold value and strong overall reliability. Discriminant validity was confirmed: all HTMT values were below 0.85; notably HTMT (PE, PU) = 0.782 and HTMT (FC, IS) = 0.714, confirming the conceptual distinctions in 2.6 at the empirical level (Fornell–Larcker criterion also satisfied).

**Table 3:** Measurement model: reliability and convergent validity (*N* = 326).

Construct	Items	Cronbach *α*	CR	AVE	Mean	SD
Performance expectancy	4	0.893	0.926	0.758	4.87	1.24
Effort expectancy	4	0.862	0.906	0.707	4.53	1.31
Social influence	4	0.851	0.899	0.691	4.71	1.18
Facilitating conditions	4	0.844	0.895	0.681	4.65	1.22
Perceived usefulness	4	0.887	0.922	0.748	4.92	1.20
Perceived ease of use	4	0.831	0.887	0.662	4.48	1.28
AI self-efficacy	5	0.902	0.927	0.717	4.39	1.35
Perceived risk	5	0.864	0.901	0.646	4.82	1.29
Institutional support	4	0.878	0.915	0.729	4.61	1.31
Attitude toward AI	4	0.912	0.935	0.782	5.04	1.22
Adoption intention	3	0.889	0.931	0.819	4.96	1.27

CR, composite reliability; AVE, average variance extracted. All outer loadings ≥ 0.70 except PR5 (0.694), retained for content validity.

### 5.4 Structural model assessment

**Figure 1:**
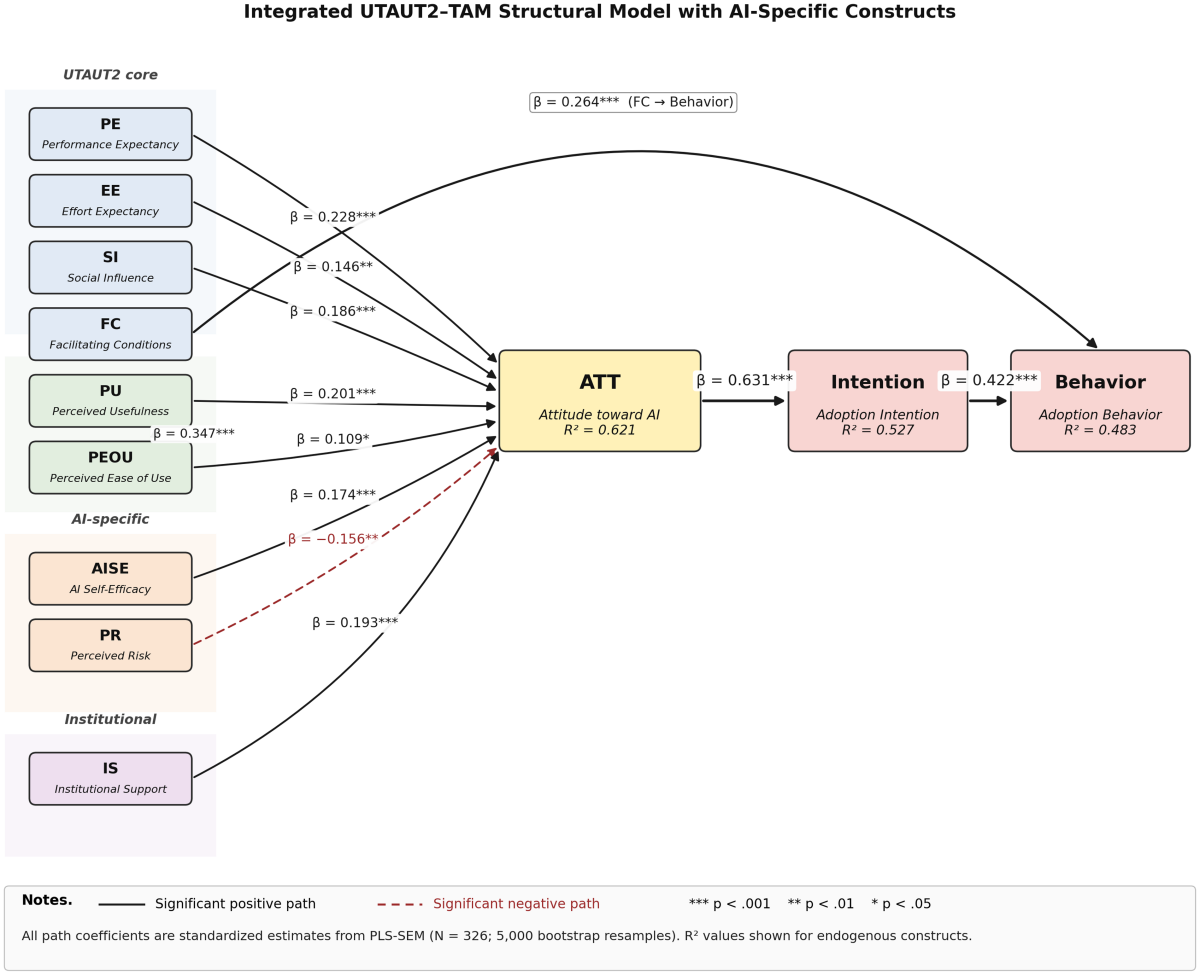
Structural model path diagram with standardized path coefficients (*β*), significance levels, and *R*^2^ values for each endogenous construct. Solid arrows = significant positive paths; dashed arrow = significant negative path (PR → ATT). ***p < 0.001; **p < 0.01; *p < 0.05.

Collinearity examination showed all inner VIF values from 1.18 to 2.67, below the threshold of 5.0 (Hair et al., 2019). Structural model results are summarized in Table 4 and visualised in Figure 1.

**Table 4:** Structural model results: path coefficients and hypothesis testing (*N* = 326).

H	Path	*β*	SE	*t*	*p*	*f* ^2^	Decision
H1	PE → ATT	0.228	0.048	4.750	<0.001	0.071	Supported
H2	EE → ATT	0.146	0.051	2.863	0.004	0.028	Supported
H3	SI → ATT	0.186	0.047	3.957	<0.001	0.049	Supported
H4	FC → Behavior	0.264	0.052	5.077	<0.001	0.083	Supported
H5	PU → ATT	0.201	0.049	4.102	<0.001	0.055	Supported
H6	PEOU → ATT	0.109	0.049	2.224	0.026	0.016	Supported
H6a	PEOU → PU	0.347	0.054	6.426	<0.001	0.138	Supported
H7	AISE → ATT	0.174	0.047	3.702	<0.001	0.042	Supported
H8	PR → ATT	−0.156	0.045	3.467	0.001	0.035	Supported
H9	IS → ATT	0.193	0.048	4.021	<0.001	0.051	Supported
H10	ATT → Intention	0.631	0.041	15.390	<0.001	0.662	Supported
H11	Intention → Behavior	0.422	0.053	7.962	<0.001	0.217	Supported

Bootstrap = 5,000 resamples. Effect sizes: *f*^2^ ≥ 0.02 small, ≥ 0.15 medium, ≥ 0.35 large (Hair et al., 2019). A path was considered supported if *p* < 0.05 and *f*^2^ ≥ 0.02.

All 12 hypothesized paths were supported (*p* < 0.05; *f*^2^ ≥ 0.016). Effect sizes varied considerably (*f*^2^ = 0.016–0.662). Performance expectancy exhibited the largest effect on attitude (*β* = 0.228, *f*^2^ = 0.071), followed by perceived usefulness (*β* = 0.201), institutional support (*β* = 0.193), social influence (*β* = 0.186), and AI self-efficacy (*β* = 0.174). Perceived risk had a significant negative association (*β* = −0.156). Effort expectancy (*β* = 0.146, *f*^2^ = 0.028) and perceived ease of use (*β* = 0.109, *f*^2^ = 0.016) were statistically significant but small in effect size; their interpretive weight should be calibrated accordingly, as PEOU’s main contribution in this dataset operates indirectly via PU (see Section 5.5). Facilitating conditions directly predicted adoption behavior (*β* = 0.264, *f*^2^ = 0.083). Faculty attitude strongly predicted adoption intention (*β* = 0.631, *f*^2^ = 0.662). The model explained 62.1% of variance in attitude (*R*^2^ = 0.621), 52.7% in adoption intention (*R*^2^ = 0.527), and 48.3% in self-reported adoption behavior (*R*^2^ = 0.483). Predictive relevance was confirmed by *Q*^2^ > 0 for all endogenous constructs (ATT *Q*^2^ = 0.461; Intention *Q*^2^ = 0.418; Behavior *Q*^2^ = 0.352).

### 5.5 Mediation analysis

The mediating role of attitude (H10) was tested using bootstrapping (5,000 resamples). All indirect effects through ATT were significant (Table 5). The largest indirect effects were: PE → ATT → Intention (indirect β = 0.144, 95% CI [0.092, 0.201]); PU → ATT → Intention (*β* = 0.127, [0.079, 0.181]); IS → ATT → Intention (*β* = 0.122, [0.074, 0.176]). Perceived risk had a significant negative indirect effect (indirect *β* = −0.098, CI [−0.148, −0.054]).

**Table 5:** Mediation analysis: indirect effects through faculty attitude (bootstrap = 5,000 resamples).

Indirect path	Indirect *β*	95% CI LL	95% CI UL	*p*	Sig.
PE → ATT → Intention	0.144	0.092	0.201	<0.001	Yes
EE → ATT → Intention	0.092	0.038	0.152	0.003	Yes
SI → ATT → Intention	0.117	0.070	0.170	<0.001	Yes
PU → ATT → Intention	0.127	0.079	0.181	<0.001	Yes
PEOU → ATT → Intention	0.069	0.028	0.114	0.010	Yes
PEOU → PU → ATT (VAF = 39.1%)	0.070	0.038	0.111	<0.001	Yes
AISE → ATT → Intention	0.110	0.063	0.165	<0.001	Yes
PR → ATT → Intention	−0.098	−0.148	−0.054	<0.001	Yes
IS → ATT → Intention	0.122	0.074	0.176	<0.001	Yes

CI, confidence interval; VAF, variance accounted for (proportion of total PEOU → ATT effect transmitted via PU); VAF 20–80% indicates partial mediation.

Within the TAM mediation chain, PEOU exerted a significant indirect effect on attitude via PU (indirect *β* = 0.070, CI [0.038, 0.111]), in addition to its direct effect. The variance accounted for (VAF = 39.1%) indicates partial mediation: both the direct PEOU → ATT route and the PU-mediated route contribute independently, consistent with the original TAM specification (Davis et al., 1989) and meta-analytic evidence (Scherer et al., 2019) (Table 6).

**Table 6:** MICOM results: measurement invariance across three focal subgroup contrasts.

Construct	Contrast A: STEM vs. Humanities—c	Contrast A: ΔM (p)	Contrast A: ΔVar (p)	Contrast B: Lecturer vs. Professor—c	Contrast B: ΔM (p)	Contrast B: ΔVar (p)	Contrast C: Low vs. High Exp—c	Contrast C: ΔM (p)	Contrast C: ΔVar (p)
PE	0.998	0.341	0.412	0.997	0.287	0.391	0.996	0.318	0.462
EE	0.997	0.408	0.374	0.998	0.312	0.428	0.997	0.291	0.384
SI	0.996	0.382	0.461	0.995	0.346	0.403	0.998	0.372	0.441
FC	0.997	0.419	0.388	0.996	0.304	0.419	0.995	0.361	0.407
PU	0.998	0.357	0.443	0.997	0.328	0.394	0.997	0.348	0.418
PEOU	0.996	0.391	0.416	0.996	0.319	0.387	0.997	0.284	0.432
AISE	0.995	0.374	0.402	0.997	0.341	0.421	0.996	0.367	0.396
PR	0.997	0.413	0.378	0.995	0.298	0.411	0.997	0.356	0.424
IS	0.997	0.367	0.431	0.998	0.312	0.398	0.996	0.341	0.415
ATT	0.998	0.342	0.419	0.997	0.327	0.406	0.997	0.363	0.428

c = compositional invariance coefficient (permutation-based; values near 1.00 support Step 2 invariance). ΔM = mean difference permutation p-value; ΔVar = variance ratio permutation p-value; p > 0.05 (two-tailed) supports Step 3 invariance. All contrasts achieved full measurement invariance (Henseler et al., 2016).

### 5.6 Measurement invariance (MICOM) results

Table 6 reports the full MICOM results for the three focal contrasts. Step 1 (configural invariance) holds by construction. Step 2 (compositional invariance) was supported for all constructs across all contrasts: permutation-based c values were at or above their 5% quantile (all *p* > 0.05 for the null of equal composition). Step 3 (equality of composite means and variances) was supported: differences in composite means (ΔM) and variances (ΔVar) fell within the respective permutation-based 95% confidence intervals for all constructs in all contrasts. Full measurement invariance is established; PLS-MGA differences in 5.7 reflect genuine structural variation rather than measurement artefacts.

### 5.7 Multi-group analysis

Following confirmed full measurement invariance (5.6), PLS-MGA was conducted across three subgroup contrasts. Disciplinary differences: the PE → ATT path was significantly stronger among STEM faculty (*β* = 0.301) than Humanities faculty (*β* = 0.142; Δ*β* = 0.159, *p* = 0.018). The PR → ATT path was significantly stronger among Humanities faculty (*β* = −0.237) than STEM faculty (*β* = −0.098; Δ*β* = 0.139, *p* = 0.024). Social influence was a stronger predictor of ATT among Arts faculty (*β* = 0.268) than STEM faculty (*β* = 0.139; Δ*β* = 0.129, *p* = 0.031).

Academic-rank differences: the IS → ATT path was significantly stronger among lecturers (*β* = 0.261) than full professors (*β* = 0.124; Δ*β* = 0.137, *p* = 0.029), suggesting junior faculty are more responsive to institutional signals. No other rank-based differences reached significance at *p* < 0.05.

Prior-technology-experience differences: faculty with high prior experience exhibited a significantly weaker EE → ATT path (*β* = 0.063, n.s.) than those with low experience (*β* = 0.228, *p* < 0.001; Δ*β* = 0.165, *p* = 0.008), replicating a core UTAUT prediction (Venkatesh et al., 2003). AISE → ATT was also stronger for low-experience faculty (*β* = 0.253) than high-experience faculty (*β* = 0.108; Δ*β* = 0.145, *p* = 0.016).

### 5.8 Qualitative findings

Codebook thematic analysis of the 22 interviews yielded six themes: five are consistent with the UTAUT2–TAM a-priori codebook (Themes 1–5) and one emerged exclusively as an inductive code not anticipated by the theoretical framework (Theme 6).

*Theme 1—Pragmatic utility as the primary driver.* Consistent with the dominance of PE and PU in the structural model, participants cited practical utility as their primary consideration. A STEM associate professor (P7): “I started using AI to generate quiz questions because it saved me hours every week. Once I saw the time savings, I was willing to invest effort in learning other features.” A Social Sciences lecturer (P12): “If it doesn’t clearly improve my teaching efficiency or student outcomes, I won’t adopt it, no matter how much the university promotes it.”

*Theme 2—Institutional support as an enabling condition, with a symbolic/substantive distinction.* Participants distinguished between symbolic institutional encouragement (policy statements, administrative rhetoric) and substantive support (dedicated training, technical assistance, time allocation). A Humanities professor (P3): “The university talks a lot about AI in education, but there’s no dedicated training for faculty in my department. I would need hands-on workshops, not just a link to an online tutorial.” An Arts lecturer (P18) who had received targeted support: “After attending the AI teaching workshop organized by the Teaching Development Center, I felt much more confident.”

*Theme 3—Professional identity and pedagogical autonomy concerns.* A recurring theme, particularly among Humanities and Arts faculty, was the perception that AI tools threatened professional identity. A Humanities associate professor (P9): “If AI can generate essay prompts and grade written assignments, what is my unique contribution as a teacher?” This theme maps onto the significant negative PR → ATT path and clarifies why this path is steeper among Humanities faculty in the MGA: the professional-identity facet of risk is more salient than the academic-integrity or data-privacy facets in humanistic disciplines.

*Theme 4—Disciplinary norms and peer influence.* Disciplinary cultures shaped adoption norms. STEM faculty described a pro-technology culture in which early adoption is expected and rewarded. A Computer Science professor (P1): “In our department, not using AI would actually be unusual.” A Philosophy lecturer (P14): “There’s an unspoken consensus in our department that critical thinking requires human guidance. AI is seen as potentially undermining deep intellectual engagement.”

*Theme 5—Evolving attitudes and the role of experience.* Several participants described attitudinal shifts from initial skepticism to cautious acceptance following positive personal experiences. A Social Sciences professor (P6): “A year ago, I was firmly against it. Then a colleague showed me how they used AI to create differentiated learning materials, and I started experimenting. My attitude has completely changed.” This is consistent with the experience-based MGA finding (Theme 5 expands on the mechanism).

*Theme 6—Compliance-driven adoption without attitudinal alignment [INDUCTIVE/EMERGENT CODE].* This theme emerged exclusively from the data—it was not part of the a-priori UTAUT2–TAM codebook—and is therefore identified as a divergent finding in the joint display (5.9). Four senior faculty members (two full professors and two associate professors with ≥ 20 years of teaching experience) reported continued active use of AI tools despite low perceived ease of use and moderate-to-high perceived risk. A Humanities professor (P15): “I find these tools frustrating and worry about what they do to students’ writing, but the department has made AI integration part of our annual review. I use them because I have to.” A Social Sciences professor (P19): “The teaching quality assessment includes AI integration indicators now. Whether I believe in this tool or not, I have to show I’m using it.” These four cases indicate that institutional mandate can drive adoption independently of the attitude-mediation mechanism (H10), a boundary condition discussed in 6.2.

### 5.9 Joint-display integration of quantitative and qualitative findings

Following Fetters et al. (2013), the two strands were integrated across four patterns: convergence, expansion, clarification, and divergence. Table 7 summarises the integration.

**Table 7:** Joint display integrating quantitative and qualitative findings (Fetters et al., 2013).

Integrated finding	Quantitative result	Qualitative theme	Integration pattern
Pragmatic utility drives adoption	PE *β* = 0.228, PU *β* = 0.201 (both *p* < 0.001; largest paths into ATT)	Theme 1—cost–benefit framing dominates adoption reasoning	Convergence—both strands consistently support utility-first adoption logic
Substantive > symbolic support	IS *β* = 0.193 (*p* < 0.001); indirect *β* = 0.122 to intention via ATT	Theme 2—participants distinguish policy rhetoric from dedicated training and workshops	Expansion—qualitative strand specifies which IS components activate the path
Humanities risk sensitivity is identity-linked	PR → ATT steeper in Humanities (*β* = −0.237) than STEM (*β* = −0.098); Δ*β* = 0.139, *p* = 0.024	Theme 3—professional-identity threat named as mechanism, distinct from integrity or privacy concerns	Clarification—qualitative strand names the specific PR facet driving the MGA difference
Disciplinary cultures shape SI weight	SI → ATT stronger in Arts (*β* = 0.268) than STEM (*β* = 0.139); Δ*β* = 0.129, *p* = 0.031	Theme 4—visible disciplinary pro- vs. anti-AI norms described by participants	Convergence—consistent across both strands
Experience attenuates EE and AISE effects	Low-exp EE *β* = 0.228; high-exp n.s. Low-exp AISE *β* = 0.253 vs. 0.108	Theme 5—attitudinal shift described as following positive first-hand experience	Convergence—temporal dimension added by qualitative strand
Compliance-driven adoption (senior faculty)	Not modelled—outside UTAUT2–TAM mechanism	Theme 6 (inductive/emergent)—institutional mandate overrides attitude among 4 senior faculty	Divergence—pathway not predictable from UTAUT2–TAM; constitutes theoretical boundary condition

## 6 Discussion

### 6.1 Key findings

The integrated UTAUT2–TAM model explains 62.1% of variance in faculty attitude (*R*^2^ = 0.621), 52.7% in adoption intention (*R*^2^ = 0.527), and 48.3% in self-reported adoption behavior (*R*^2^ = 0.483). These *R*^2^ values are higher than those typically reported in standalone TAM-based studies (mean *R*^2^ ≈ 0.40 for behavioral intention; Scherer et al., 2019) and in UTAUT-based studies in education (0.35–0.48, Khechine et al., 2020). This comparison is against published meta-analytic benchmarks from different samples and contexts; formal within-dataset comparison—fitting TAM-only and UTAUT2-only specifications—was not conducted and remains a direction for future research. The *R*^2^ for attitude (0.621) is the more substantive explanatory achievement: it reflects nine theoretically-grounded predictors and is less susceptible to the near-tautology concern associated with the attitude → intention path.

All 12 hypotheses were supported, though effect sizes varied considerably (*f*^2^ = 0.016–0.662). Two paths—PEOU → ATT (*β* = 0.109, *f*^2^ = 0.016) and EE → ATT (*β* = 0.146, *f*^2^ = 0.028)—achieved statistical significance but small effect sizes; they should be interpreted cautiously as minor contributors to attitude formation, with PEOU’s primary contribution running indirectly through PU (VAF = 39.1%).

### 6.2 Theoretical implications

First, the strong mediating role of faculty attitude (*β* = 0.631, *f*^2^ = 0.662) supports Dwivedi et al.'s (2019) argument for reintroducing attitude into UTAUT-based models. For AI adoption—where faculty hold complex, often ambivalent evaluations shaped by instrumental, experiential, and risk considerations—attitude functions not as a redundant mediator but as the psychological mechanism through which diverse cognitive appraisals are integrated into a coherent evaluative orientation (Ajzen, 1991; Davis, 1989). This is consistent with Wang et al.'s (2021) demonstration that attitude plays a central mediating role in faculty AI adoption in Chinese and Taiwanese higher education.

Second, the significant effects of the AI-specific constructs—AI self-efficacy (*β* = 0.174) and perceived risk (*β* = −0.156)—demonstrate that traditional technology-acceptance constructs alone are insufficient for explaining faculty adoption of AI. AISE, extending computer self-efficacy (Compeau and Higgins, 1995), captures the confidence demands of navigating opaque algorithmic systems. PR, drawing on Featherman and Pavlou's (2003) risk framework and the AI-specific taxonomy of Chiu et al. (2023), captures faculty concerns that are distinctive to AI tools: threats to academic integrity, pedagogical autonomy, and algorithmic reliability.

Third, the MICOM-validated multi-group analysis reveals that the adoption process is not uniform across disciplinary, rank-based, and experiential subgroups. The stronger PE → ATT path among STEM faculty and the stronger PR → ATT path among Humanities faculty indicate that disciplinary epistemologies and professional cultures condition the weighting of adoption factors in systematic ways (Teo, 2011). The attenuation of EE’s influence with prior technology experience replicates a core UTAUT prediction (Venkatesh et al., 2003).

Fourth, the PEOU → PU → ATT chain (VAF = 39.1%) replicates a foundational TAM relationship in the AI-teaching context (Davis et al., 1989). Faculty who find AI tools intuitive are more likely to recognise their task-level utility, which in turn shapes a favorable attitude. This cumulative pathway underscores the importance of user-interface design and structured onboarding.

Fifth, and most consequentially for theory, the inductive Theme 6—compliance-driven adoption without attitudinal alignment—identifies a boundary condition for the attitude-mediation mechanism (H10). When senior faculty adopt AI tools primarily to satisfy departmental review requirements or teaching-quality indicators, the causal chain prescribed by UTAUT2–TAM (antecedents → attitude → intention → behavior) is bypassed: behavior is driven by institutional mandate, not by attitudinal orientation. When individual faculty comply with AI-integration requirements despite unfavorable attitudes, the aggregate pattern constitutes organizational-level coercive isomorphism in the sense of Meyer and Rowan (1977) and DiMaggio and Powell (1983): conformity is driven by legitimacy demands—the need to appear aligned with institutional expectations—rather than by efficiency beliefs or genuine evaluative acceptance. The individual-to-organizational bridge is straightforward: when a sufficient number of faculty exhibit compliance-driven adoption, the institution can report high aggregate AI-integration rates while the actual attitudinal infrastructure for sustained, pedagogically meaningful use remains fragile. This boundary condition suggests that the attitude-mediation mechanism operates conditionally on voluntariness: it is robust under low-to-moderate institutional pressure but attenuates when mandates are sufficiently strong to override attitudinal determinants. Future research should operationalize institutional-pressure intensity as a formal moderator of the H10 path—for example, via a perceived-mandate scale that distinguishes between recommendation, encouragement, and formal evaluation requirement. The finding also points toward a refinement of the PR construct: the professional-identity facet identified in Theme 3 should be incorporated alongside the five existing facets in future AI-adoption scales, as the Humanities subgroup difference in MGA appears attributable specifically to identity threat rather than integrity or privacy concerns.

### 6.3 Practical implications

Four institutional levers follow from the findings.

- Foreground concrete teaching benefits. The dominance of PE and PU—reinforced by Theme 1—indicates that adoption promotion should foreground documented efficiency gains rather than top-down mandates. Peer-led showcases, case studies of specific classroom applications, and visible early-adopter exemplars are more effective than general encouragement.
- Move institutional support from rhetoric to substance. The significant IS path (*β* = 0.193) and the qualitative symbolic/substantive distinction call for systemic rather than rhetorical approaches: dedicated AI teaching-support units, protected time for experimentation, discipline-specific training, and recognition of AI-enhanced teaching in tenure and promotion criteria.
- Proactively govern risk, particularly for the Humanities. The stronger PR effect among Humanities faculty—attributable to professional-identity threat—requires explicit risk-governance: transparent guidelines on appropriate AI use, academic-integrity policies for AI-generated content, faculty communities of practice, and assurance that experimental AI use will not be penalized in teaching evaluations.
- Differentiate professional development by experience and discipline. The stronger AISE effect among low-experience faculty indicates that hands-on workshops and mentorship from early adopters are critical for building confidence. Programmes should be differentiated by discipline: STEM faculty may benefit from advanced technical training, whereas Humanities and Arts faculty need support that addresses both technical skills and pedagogical-integration strategies, including explicit engagement with professional-identity concerns.

### 6.4 Interpretive caveats (statistical model)

Two model-internal caveats should be noted when interpreting the structural results.

First, the attitude → adoption intention path (*β* = 0.631, *f*^2^ = 0.662) is unusually large. Part of this magnitude likely reflects conceptual and measurement overlap at the latent level: an evaluative orientation toward AI (ATT) and a stated intention to use AI (AI-Intent) are proximate constructs that share conceptual territory. This does not invalidate the mediation findings—attitude remains the substantive psychological mechanism—but it does mean that the *R*^2^ for adoption intention (0.527) should be read in conjunction with the *R*^2^ for attitude (0.621) as the primary explanatory benchmark. Replication with more sharply differentiated item wordings would strengthen inference on this specific path.

Second, the claim that the integrated UTAUT2–TAM model is superior to standalone TAM or UTAUT2 rests on comparison against meta-analytic benchmarks from different studies and contexts (Scherer et al., 2019; Khechine et al., 2020), not on within-dataset rival-model testing. Fitting TAM-only and UTAUT2-only specifications against the full model on the present dataset and comparing information criteria remains an important direction for future replication work.

### 6.5 Limitations and future research

Five limitations should be acknowledged. First, the cross-sectional design limits causal inference; longitudinal tracking over multiple semesters would enable stronger temporal claims about adoption trajectories (Rogers, 2003; Dwivedi et al., 2019). Second, single-institution sampling at Shanghai University limits generalizability to teaching-focused universities, vocational colleges, and institutions in other regional contexts; multi-institution replication is a priority for future work (Teo, 2011). Third, both the predictors and the behavioral outcome rely on self-report within the same instrument. The dependent variable ‘self-reported adoption behavior’ captures faculty perceptions of their own usage rather than objectively logged platform behavior; it is therefore more accurately understood as a behavioral intention at a near-term time horizon than as verified behavior. Future studies should incorporate platform log data or structured teaching-observation data to complement self-report and reduce residual common-method variance (Kock, 2015; Hair et al., 2019). Fourth, the model omits potentially relevant variables such as pedagogical beliefs, departmental governance structures, student expectations, and differences among types of AI tools (generative vs. analytical; general-purpose vs. domain-specific); these warrant investigation in extended models (Zawacki-Richter et al., 2019; Kasneci et al., 2023). Fifth, the compliance-driven adoption pathway (Theme 6) was identified from four participants and is therefore hypothesis-generating rather than conclusively established; future research should operationalize institutional-pressure intensity as a formal moderator and test its conditioning effect on the attitude-mediation path in larger samples with sufficient variance in voluntariness of AI use.

## 7 Conclusion

This study tested an integrated UTAUT2–TAM framework for faculty adoption of AI-assisted teaching tools at Shanghai University. A mixed-methods sequential explanatory design combining a quantitative PLS-SEM analysis (*N* = 326) with codebook thematic analysis of 22 interviews showed that the integrated model explains 62.1% of the variance in faculty attitude, 52.7% in adoption intention, and 48.3% in self-reported adoption behavior.

All 12 hypotheses were supported, with varying effect sizes. Performance expectancy, perceived usefulness, institutional support, social influence, and AI self-efficacy were the strongest positive correlates of faculty attitude; perceived risk was a significant negative correlate; perceived ease of use contributed both directly and indirectly via perceived usefulness (VAF = 39.1%). Faculty attitude strongly mediated between antecedents and adoption intention. Following full MICOM-validated measurement invariance, multi-group analysis revealed discipline-, rank-, and experience-based differences in path strengths. Qualitative findings converged with the quantitative pattern on pragmatic utility and institutional support; clarified the identity-threat mechanism behind Humanities faculty’s stronger risk sensitivity; and—through the inductive Theme 6—identified a compliance-driven adoption pathway among senior faculty that constitutes a boundary condition for the attitude-mediation mechanism not captured by UTAUT2–TAM.

Two specific theoretical advances emerge. The MICOM-validated MGA demonstrates that disciplinary culture systematically conditions AI adoption pathways—a finding that requires measurement-invariance verification to be credible and that prior studies have not provided. The boundary condition identified by Theme 6 frames institutional-pressure intensity as a candidate moderator of the attitude-mediation mechanism, opening a theoretically consequential line of inquiry into conditional TAM/UTAUT2 processes. Practically, the findings identify four institutional levers: concrete teaching-benefit showcases, substantive institutional support, proactive risk governance, and discipline-differentiated professional development. As AI continues to reshape higher-education teaching in China and internationally, these findings offer both a validated framework and an empirical evidence base for administrators and researchers designing AI-integration policy.

## Data Availability

The original contributions presented in the study are included in the article and supplementary material. Further inquiries, including requests for de-identified supporting data where ethically permissible, can be directed to the corresponding author.

## References

[ref1] Ajzen I. (1991). The theory of planned behavior. Organ. Behav. Hum. Decis. Process. 50, 179–211. doi: 10.1016/0749-5978(91)90020-T

[ref2] An X. Chai C. S. Li Y. Zhou Y. Shen X. Zheng C. . (2023). Modeling English teachers' behavioral intention to use artificial intelligence in middle schools. Educ. Inf. Technol. 28, 5187–5208. doi: 10.1007/s10639-022-11286-z

[ref3] Bagozzi R. P. (2007). The legacy of the technology acceptance model and a proposal for a paradigm shift. J. Assoc. Inf. Syst. 8, 244–254. doi: 10.17705/1jais.00122

[ref4] Baidoo-Anu D. Ansah L. O. (2023). Education in the era of generative artificial intelligence (AI): understanding the potential benefits of ChatGPT in promoting teaching and learning. J. AI 7, 52–62. doi: 10.61969/jai.1337500

[ref5] Bandura A. (1986). Social Foundations of Thought and Action: A Social Cognitive Theory. Englewood Cliffs, NJ: Prentice-Hall.

[ref6] Boyatzis R. E. (1998). Transforming Qualitative Information: Thematic Analysis and Code Development. Thousand Oaks, CA: SAGE.

[ref7] Braun V. Clarke V. (2019). Reflecting on reflexive thematic analysis. Qual. Res. Sport, Exerc. Health 11, 589–597. doi: 10.1080/2159676X.2019.1628806

[ref8] Braun V. Clarke V. (2021). One size fits all? What counts as quality practice in (reflexive) thematic analysis? Qual. Res. Psychol. 18, 328–352. doi: 10.1080/14780887.2020.1769238

[ref9] Brislin R. W. (1970). Back-translation for cross-cultural research. J. Cross-Cult. Psychol. 1, 185–216. doi: 10.1177/135910457000100301

[ref10] Chan C. K. Y. Hu W. (2023). Students' voices on generative AI: perceptions, benefits, and challenges in higher education. Int. J. Educ. Technol. High. Educ. 20:43. doi: 10.1186/s41239-023-00411-8

[ref11] Chatterjee S. Bhattacharjee K. K. (2020). Adoption of artificial intelligence in higher education: a quantitative analysis using structural equation modelling. Educ. Inf. Technol. 25, 3443–3463. doi: 10.1007/s10639-020-10159-7

[ref12] Chiu T. K. F. Xia Q. Zhou X. Chai C. S. Cheng M. (2023). Systematic literature review on opportunities, challenges, and future research recommendations of artificial intelligence in education. Comput. Educ. Artif. Intell. 4:100118. doi: 10.1016/j.caeai.2022.100118

[ref13] Compeau D. R. Higgins C. A. (1995). Computer self-efficacy: development of a measure and initial test. MIS Q. 19, 189–211. doi: 10.2307/249688

[ref14] Creswell J. W. Plano Clark V. L. (2018). Designing and Conducting Mixed Methods Research. 3rd Edn. Thousand Oaks, CA: SAGE.

[ref15] Crompton H. Burke D. (2023). Artificial intelligence in higher education: the state of the field. Int. J. Educ. Technol. High. Educ. 20:22. doi: 10.1186/s41239-023-00392-8

[ref16] Davis F. D. (1989). Perceived usefulness, perceived ease of use, and user acceptance of information technology. MIS Q. 13, 319–340. doi: 10.2307/249008

[ref17] Davis F. D. Bagozzi R. P. Warshaw P. R. (1989). User acceptance of computer technology: a comparison of two theoretical models. Manag. Sci. 35, 982–1003. doi: 10.1287/mnsc.35.8.982

[ref18] DiMaggio P. J. Powell W. W. (1983). The iron cage revisited: institutional isomorphism and collective rationality in organizational fields. Am. Sociol. Rev. 48, 147–160. doi: 10.2307/2095101

[ref19] Dwivedi Y. K. Rana N. P. Jeyaraj A. Clement M. Williams M. D. (2019). Re-examining the unified theory of acceptance and use of technology (UTAUT): towards a revised theoretical model. Inf. Syst. Front. 21, 719–734. doi: 10.1007/s10796-017-9774-y

[ref20] Featherman M. S. Pavlou P. A. (2003). Predicting e-services adoption: a perceived risk facets perspective. Int. J. Hum. Comput. Stud. 59, 451–474. doi: 10.1016/S1071-5819(03)00111-3

[ref21] Fetters M. D. Curry L. A. Creswell J. W. (2013). Achieving integration in mixed methods designs — principles and practices. Health Serv. Res. 48, 2134–2156. doi: 10.1111/1475-6773.12117, 24279835 PMC4097839

[ref22] Granić A. Marangunić N. (2019). Technology acceptance model in educational context: a systematic literature review. Br. J. Educ. Technol. 50, 2572–2593. doi: 10.1111/bjet.12864

[ref23] Guest G. MacQueen K. M. Namey E. E. (2012). Applied Thematic Analysis. Thousand Oaks, CA: SAGE.

[ref24] Hair J. F. Risher J. J. Sarstedt M. Ringle C. M. (2019). When to use and how to report the results of PLS-SEM. Eur. Bus. Rev. 31, 2–24. doi: 10.1108/EBR-11-2018-0203

[ref25] Henseler J. Ringle C. M. Sarstedt M. (2016). Testing measurement invariance of composites using partial least squares. Int. Mark. Rev. 33, 405–431. doi: 10.1108/IMR-09-2014-0304

[ref26] Holmes W. Bialik M. Fadel C. (2022). Artificial Intelligence in Education: Promises and Implications for Teaching and Learning. Boston, MA: Center for Curriculum Redesign.

[ref27] Kasneci E. Seßler K. Küchemann S. Bannert M. Dementieva D. Fischer F. . (2023). ChatGPT for good? On opportunities and challenges of large language models for education. Learn. Individ. Differ. 103:102274. doi: 10.1016/j.lindif.2023.102274

[ref28] Khechine H. Raymond B. Augier M. (2020). The adoption of a social learning system: intrinsic value in the UTAUT model. Br. J. Educ. Technol. 51, 2306–2325. doi: 10.1111/bjet.12905

[ref29] King N. (2012). “Doing template analysis,” in Qualitative Organizational Research: Core Methods and Current Challenges, eds. Symon G. Cassell C. (London: SAGE), 426–450.

[ref30] Kock N. (2015). Common method bias in PLS-SEM: a full collinearity assessment approach. Int. J. E-collab. 11, 1–10. doi: 10.4018/ijec.2015100101

[ref31] Landis J. R. Koch G. G. (1977). The measurement of observer agreement for categorical data. Biometrics 33, 159–174. doi: 10.2307/2529310, 843571

[ref32] Meyer J. W. Rowan B. (1977). Institutionalized organizations: formal structure as myth and ceremony. Am. J. Sociol. 83, 340–363. doi: 10.1086/226550

[ref33] Ministry of Education of the PRC (2018) Education informatization 2.0 action plan [in Chinese]. Available online at: http://www.moe.gov.cn/srcsite/A16/s3342/201804/t20180425_334188.html (Accessed May 14, 2026).

[ref34] Moorhouse B. L. Yeo M. A. Wan Y. (2023). Generative AI tools and assessment: guidelines of the world's top-ranking universities. Comput. Educ. Open 5:100151. doi: 10.1016/j.caeo.2023.100151

[ref35] Nunnally J. C. (1978). Psychometric Theory. 2nd Edn. New York, NY: McGraw-Hill.

[ref36] Podsakoff P. M. MacKenzie S. B. Lee J.-Y. Podsakoff N. P. (2003). Common method biases in behavioral research: a critical review of the literature and recommended remedies. J. Appl. Psychol. 88, 879–903. doi: 10.1037/0021-9010.88.5.879, 14516251

[ref37] Ringle C. M. Wende S. Becker J.-M. (2022) SmartPLS 4 SmartPLS GmbH. Available online at: https://www.smartpls.com (Accessed May 14, 2026).

[ref38] Rogers E. M. (2003). Diffusion of Innovations. 5th Edn. New York, NY: Free Press.

[ref39] Scherer R. Siddiq F. Tondeur J. (2019). The technology acceptance model (TAM): a meta-analytic structural equation modeling approach to explaining teachers' adoption of digital technology in education. Comput. Educ. 128, 13–35. doi: 10.1016/j.compedu.2018.09.009

[ref40] Shanghai Municipal Education Commission (2021). Action Plan for AI-Enabled Education Innovation in Shanghai (2021–2025) [in Chinese]. Shanghai: Shanghai Municipal Government.

[ref41] Shanghai University (2023). Annual Report on Educational Informatization and smart campus Construction [in Chinese]. Shanghai: Shanghai University Press.

[ref42] State Council of the PRC (2017) New generation artificial intelligence development plan [in Chinese]. Available online at: http://www.gov.cn/zhengce/content/2017-07/20/content_5211996.htm (Accessed May 14, 2026).

[ref43] Straub E. T. (2009). Understanding technology adoption: theory and future directions for informal learning. Rev. Educ. Res. 79, 625–649. doi: 10.3102/0034654308325896

[ref44] Tashakkori A. Teddlie C. (2010). SAGE Handbook of Mixed Methods in Social and Behavioral Research. 2nd Edn. Thousand Oaks, CA: SAGE.

[ref45] Teo T. (2011). Factors influencing teachers' intention to use technology: model development and test. Comput. Educ. 57, 2432–2440. doi: 10.1016/j.compedu.2011.06.008

[ref46] Venkatesh V. Bala H. (2008). Technology acceptance model 3 and a research agenda on interventions. Decis. Sci. 39, 273–315. doi: 10.1111/j.1540-5915.2008.00192.x

[ref47] Venkatesh V. Davis F. D. (2000). A theoretical extension of the technology acceptance model: four longitudinal field studies. Manag. Sci. 46, 186–204. doi: 10.1287/mnsc.46.2.186.11926

[ref48] Venkatesh V. Morris M. G. Davis G. B. Davis F. D. (2003). User acceptance of information technology: toward a unified view. MIS Q. 27, 425–478. doi: 10.2307/30036540

[ref49] Venkatesh V. Thong J. Y. L. Xu X. (2012). Consumer acceptance and use of information technology: extending the unified theory of acceptance and use of technology. MIS Q. 36, 157–178. doi: 10.2307/41410412

[ref50] Wang T. Cheng E. C. K. (2021). An investigation of barriers to Hong Kong K-12 schools incorporating artificial intelligence in education. Comput. Educ. Artif. Intell. 2:100031. doi: 10.1016/j.caeai.2021.100031

[ref51] Wang Y. Liu C. Tu Y.-F. (2021) Factors affecting the adoption of AI-based applications in higher education: an analysis of teachers' perspectives using structural equation modeling Educ. Technol. Soc. 24 116–129. Available online at: https://www.jstor.org/stable/27032860 (Accessed May 14, 2026).

[ref52] Zawacki-Richter O. Marín V. I. Bond M. Gouverneur F. (2019). Systematic review of research on artificial intelligence applications in higher education — where are the educators? Int. J. Educ. Technol. High. Educ. 16:39. doi: 10.1186/s41239-019-0171-0

